# Bristol Medico-Chirurgical Society

**Published:** 1898-12

**Authors:** 


### MEETINGS OF SOCIETIES.

### Bristol Medico-Chirurgical Society.

*Annual Meeting, October 12th,* 1898.

Dr. J. E. Shaw, President, in the Chair.

After a vote of thanks to the retiring President (Dr. J. E. Shaw) had been unanimously passed, Dr. R. Roxburgh (the President-elect) took the Chair, and gave his introductory address (see page 285), for which a cordial vote of thanks was given him.

The Honorary Secretary (Mr. J. Paul Bush) read his Annual Report, in which he stated that the Members of the Society now numbered 137. He referred in feeling terms to the loss the members had sustained by the death of Mr. Augustin Prichard and Dr. Henry Marshall.

Dr. B. M. H. Rogers in his Report as Editorial Secretary asked members of the Society to assist him in getting new subscribers for the *Journal*, for there were still a large number of medical men in Bristol and the neighbourhood who neither belonged to the Society nor subscribed to the *Journal*, and he felt that more could be done by individual help than by the usual method of sending out reply post-cards.

The Honorary Librarian (Mr. L. M. Griffiths) read his Annual Report (see page 369).

With the exception of the words from "as it would" to "its guardian," in the last paragraph but two in the Honorary Librarian's Report (see page 373), all these Reports were adopted, and the readers of them thanked for their services.

The following officers were chosen for the ensuing year:—President-elect; Mr. W. H. Harsant: Honorary Secretary; Mr. J. Paul Bush: Honorary Librarian; Mr. L. M. Griffiths: Members of Committee; Dr. J. Michell Clarke, Mr. J. Dacre, Dr. D. S. Davies, Dr. B. M. H. Rogers, Mr. G. Munro Smith, and Dr. H. Waldo: Representatives of the Society on the Committee of the Bristol Medical Library; Mr. L. M. Griffiths, Dr. G. Parker, and Mr. J. Taylor.

---

*November 9th,* 1898.

Dr. R. Roxburgh, President, in the Chair.

Mr. T. Carwardine showed a patient and the cyst removed in the case of a hydatid of the liver, and the liver and cyst of another case. The first patient was operated on in June last. He had shown signs of a hydatid of the liver for a week, and, owing to sudden pain and collapse, an immediate operation was performed. The fluid, amounting to fifty ounces, was drawn off, and the whole sterile mother cyst-wall removed at the time of operation. The patient left the Bristol Royal Infirmary six and a-half weeks after operation with a small sinus, which has since healed. Attention was drawn to the danger of septicæmia if the cyst be not removed at the time of operation on the one hand, and the liability to fatal hemorrhage by its immediate removal on the other hand. The second specimen was a large pendulous hydatid of the liver, which was very freely movable in the abdomen and simulated a hydronephrotic kidney. A full report of the case is published in vol. xlix. of the *Transactions of the Pathological Society of London*, p. 130.

Dr. E. H. E. Stack (for Dr. Shaw) showed some specimens of brain tumour. (1) Glioma of Brain from a woman of 26, whose symptoms began six months previously with headache and vomiting. On admission to the Bristol Royal Infirmary she was blind, having double optic neuritis, and was about seven months pregnant. She gave a very intelligent history of her condition; she had very severe headaches and persistent vomiting; she soon fell into a torpid sort of condition, and there was an increase of albumen in the urine, urea was diminished to .7 per cent. One day she was delivered of an eight-months child, and next day was much better and the albumin diminished, the urea increased and remained high till death. Some time after this she had a convulsion, which was repeated next day, and developed right facial paralysis. She still answered questions; a few days later she died. At the necropsy there was found a very large glio-sarcoma, situated in the right hemisphere, in the lower parietal, and extending into the frontal region to within half-an-inch of the frontal pole, the size being larger than a bantam's egg. The case is interesting (*a*) because it to some extent simulated a renal case, (*b*) because of the very large tumour without any paralysis except of the seventh nerve just before death. (2) Sarcoma of Brain, from a man of 24. For six months he had diplopia, vomiting, and headache. On admission he staggered a little on walking, his mental condition was good, there was no facial paresis, but he had slight hemiplegia and hemianæsthesia. There was double optic neuritis, with hemorrhages; vision was good, but the movements of the eyes were defective, showing interference with the third and sixth nerves on both sides. The diagnosis made then was a tumour in the interpeduncular region. He gradually became more lethargic and died two months after admission. A rounded encapsuled tumour was found in the right crus which bulged into the interpeduncular region and also extended into the posterior part of the optic thalamus. Section shows a mixed-celled sarcoma, large, small, and spindle cells. (3) Gumma, from a man aged 42, who had had syphilis twenty years before. He walked to the Infirmary, staggering a little, but with good intellect. For fourteen days he had had headache in the frontal region. On admission, he had double optic neuritis, very slight left hemiplegia, right facial paralysis with spasmodic contraction of right facial muscles. No anæsthesia. He very rapidly fell into a state resembling the second stage of general paralysis, and in six weeks he died. A circumscribed tumour, the size of a pigeon's egg, was found adherent to the pia mater and extending inwards from this at the base of the ascending frontal convolution on the right side.—Dr. Michell Clarke said that the first case opened up the very interesting question as to the occurrence in tumours of the frontal lobes of mental symptoms in a more pronounced form than in tumours in other parts of the brain. He agreed with those who attributed diagnostic importance to the presence of marked signs of mental disorder early in the illness, together with the general signs of an intracranial tumour, as pointing to the præfrontal area as the probable seat of the growth; this received confirmation if, later, hemiparesis appeared, or, in a tumour of the left hemisphere, aphasia. A peculiar hilarious mental state was sometimes observed in frontal tumours. Further investigations were necessary as to whether points of distinction could be established between growths in the cortex and those in the white matter of the frontal lobes, apart, of course, from signs of aphasia. The importance of an early and accurate diagnosis of tumours in this situation lay in their accessibility to operative interference.—Mr. ROGER WILLIAMS, remarking on the absence of any symptoms in one of the cases, said he thought it would tend to facilitate the diagnosis of intracranial tumours if it were clearly determined precisely in what localities tumours might be found without producing any noticeable symptoms. His attention had been specially directed to this subject, in connection with studying secondary tumours due to cancer of the breast. In this disease intracranial dissemination was much commoner than was generally believed; but it was the exception rather than the rule for these secondary tumours to cause noticeable symptoms. In many hospitals in this country it was unusual to open the skull as a matter of *post-mortem* routine, consequently English statistics showed a much smaller percentage of intracranial metastases than the best German statistics.

Mr. T. CARWARDINE showed a specimen of double hydrosalpinx from a patient, a midwife, who was sent to him for the purpose of hysterectomy for supposed fibroids, but who was too ill for any major operation. Six months previously she got œdema of the left leg. After admission this extended to the right leg and was followed by subcutaneous hemorrhage in the left groin, hemiplegia involving the left face, arm, and leg, and later on by gangrene of both feet. *Post mortem* an enormous hydrosalpinx was found on each side of the uterus, containing a coffee-ground-like fluid, the right rising into the lumbar region and the left being tightly coiled up behind the uterus, and there was a large thrombus in the inferior vena cava. There was cancer of the left iliac glands and peritoneum, with nodules in the liver, and a deposit in the thyroid gland. She also had well-marked vegetative endocarditis, an embolus in the right middle cerebral artery which Dr. Symes reported to contain micrococci, and infarcts in spleen and kidneys. The primary carcinoma had not yet been discovered.—Dr. J. O. SYMES said in this case he was able to make only coverslip preparations, as preservative fluid had been added to the specimens. The cocci in the cerebral embolus were apparently staphylococci.

Mr. PAUL BUSH showed: (1) A portion of a large tumour from the axilla of a man aged 39. To remove this completely he had to divide both pectoral muscles, as the mass extended up under the clavicle. On clearing out the growth from around the vessels, the axillary vein was divided; this vessel was ligatured above and below the affected area, and gave no after-trouble. The patient had been seen frequently since the operation, and there were no signs of any recurrence of the tumour, the movement of the arm was perfect. Under the microscope the tumour appeared to contain a large number of giant cells, and he considered it to be of tubercular origin. (2) A series of enlarged bursæ removed from patients suffering from housemaid's knee. He advocated complete removal of the sac under strict antiseptic precautions in the majority of cases, as being the most successful form of treatment, as recovery was very rapid and there was no possibility of any return of the trouble.—Mr. C. A. MORTON had no objection to excise a portion of the axillary vein when it was found infiltrated with malignant growth in clearing out cancerous glands from the axilla. He had seen no bad effects from such a procedure in his own practice.—Mr. F. LACE considered that the glands in Mr. Bush's case very much resembled syphilitic ones, and drew attention to the marked resemblance between some of the glands and a gummatous testis.

—Mr. W. H. Harsant said that in cases of recent bursitis he had generally found it quite sufficient to make a small incision without an anæsthetic, and to insert a drainage-tube for forty-eight hours. At the end of a week the patient could return to work. In chronic bursitis, with thickened and fibrous walls, it was no doubt necessary to excise the whole bursa in order to effect a complete cure.—Mr. Roger Williams had often seen portions of axillary vein excised in operations without any ill consequences other than temporary œdema. Ligature of the artery he considered more dangerous; but generally no ill result ensued. Contemporary ligature of artery and vein was risky, but had been done without ill result. The great nerve cords did not admit of any surgical interference in these cases. With regard to the treatment of the prepatellar bursæ, it was desirable to recognise the precise anatomical position of the bursa affected. He had found on dissection four prepatellar bursæ: (1) Between skin and subcutaneous tissue. (2) Between subcutaneous tissue and fascia lata. (3) Between fascia lata and quadriceps expansion (the commonest seat). (4) Beneath quadriceps expansion, between it and patella. Bursæ superficial to fascia lata seldom required extirpation.—Mr. W. M. Barclay, in reference to the question of excision of a portion of the axillary vein, remarked that the determining point was whether, in the case of carcinomatous or tubercular glands, the mass was so intimately blended with the vein as to make separation dangerous, either immediately from entrance of air, or remotely by preventing complete removal of a cancerous growth. It seemed to him better surgery to excise a part of the vein, and so do the utmost possible to get rid of the entire growth, than to run the almost certain chance of recurrence after a dangerous and elaborate dissection. He had excised parts of the axillary and internal jugular veins without any ill effects.—The President remarked that the views expressed by Mr. Harsant coincided with those stated by himself in a paper which he published on the treatment of chronic bursitis. There he had advocated a simple antiseptic incision with a tenotomy knife, and drainage by means of a skein of carbolised catgut (which was quite sufficient for non-purulent serous fluid); an antiseptic dressing being applied, and the limb placed on a posterior splint. At the end of a week the dressing was changed, and healing was generally found to be complete, the portion of catgut inside being cut off by the advancing cicatricial tissue from the dry portion left outside. Of course, this simple method would be useless in indurated bursæ.

Dr. B. M. H. Rogers showed some specimens of blood from a boy aged 11½ years, suffering from leucocythæmia. He came to the Children's Hospital at the end of September from the Bradford-on-Avon Workhouse; and no history of his case has been obtainable. The spleen was found to be very large, reaching to the pelvis below and almost to the umbilicus. He also suffered from optic neuritis, and is nearly blind. The number of white corpuscles on admission was 1,000,000, the red 1,920,000 per c.mm.; and on November 2nd, 1,270,000 red to 635,000 white; about in each case two to one, the difference in the numbers at the observations being due to the fact that the counting was done by different persons, or that the boy is going downhill. He has been on bone-marrow tabloids, but no improvement has taken place. Under the microscope the usual appearance could be observed, the great increase in white corpuscles (myelocytes), presence of eosinophile cells, and nucleated red corpuscles. The specimens were stained with eosine and hæmatoxylin, or eosine and Löffler's blue.

[1] *Edinb. M. J.*, 1877–78, xxiii. 498.

Dr. E. H. E. Stack showed a specimen of aneurysm of the vena cava. This interesting and uncommon specimen was very kindly sent to the Royal Infirmary by Dr. Blatchford. It was taken from a man of 55, who had general paralysis, and died of chronic nephritis at Fishponds Asylum. He had had some swelling of the legs, but no further symptoms which would have suggested obstruction of the vena cava. The specimen shows thrombosis of the whole inferior vena cava extending into the renal lumbar and common iliac veins. The upper part of the vena cava has evidently been obliterated for some time, as it is only a fibrous cord, and there is a curious abnormality in the arrangement of its tributaries. Between the obstruction and the upper lumbar veins at their junction the vessel is dilated to the size of a golf-ball, and distended by a firm bloodclot. —Professor E. Fawcett pointed out that as a result of the plugging of the inferior vena cava the communication existing often between the left renal vein and the left inferior azygos had become enlarged to carry on the circulation, the condition being similar to persistence of the left cardinal vein.

Dr. H. L. Ormerod read a paper on an acute case of ulcerative endocarditis. (This, and the discussion which followed it, will be printed in a future number.)

J. Paul Bush, *Hon. Sec.*

